# What can we learn about selective attention processes in individuals with chronic pain using reaction time tasks? A systematic review and meta-analysis

**DOI:** 10.1097/j.pain.0000000000002885

**Published:** 2023-04-12

**Authors:** Ahmad N. Abudoush, Amna Noureen, Maria Panagioti, Ellen Poliakoff, Dimitri M.L. Van Ryckeghem, Alexander Hodkinson, Nusrat Husain

**Affiliations:** a School of Health Sciences, Faculty of Biology Medicine and Health Sciences, The University of Manchester, Manchester, United Kingdom; b Department of Psychology, School of Arts, The University of Jordan, Amman, Jordan; c Department of Applied Psychology, National University of Modern Languages, Islamabad, Pakistan; d Faculty of Psychology and Neuroscience, Maastricht University, Maastricht, the Netherlands; e Department of Experimental-Clinical and Health Psychology, Ghent University, Ghent, Belgium; f Department of Behavioural and Cognitive Sciences, University of Luxembourg, Esch-sur-Alzette, Luxembourg

**Keywords:** Chronic pain, Selective attention, Systematic review, Attention processes, Stroop, Dot-probe

## Abstract

Information-processing biases such as attentional, interpretation, and memory biases are believed to play a role in exacerbating and maintaining chronic pain (CP). Evidence suggests that individuals with CP show attentional bias toward pain-related information. However, the selective attentional processes that underpin this bias are not always well outlined in the literature. To improve current understanding, a systematic review was performed using a descriptive synthesis of reaction time-based studies. A random-effects meta-analysis was added to explore whether the results of previous meta-analyses would be confirmed using studies with a larger sample size. For this review, 2008 studies were screened from 4 databases, of which 34 (participant n = 3154) were included in the review and a subset of 15 (participant n = 1339) were included in the meta-analysis. Review results were summarised by producing a descriptive synthesis for all studies. Meta-analysis results indicated a mild significant attentional bias toward sensory pain-related information (k = 15, g = 0.28, 95% CI [0.16, 0.39], I^2^ = 43.2%, *P* = 0.038), and preliminary evidence of significant moderate bias towards affective pain-related information (k = 3, g = 0.48, 95% CI [0.23, 0.72], I^2^ = 7.1%, *P* = 0.341) for CP groups compared with control groups. We explored the main tasks, stimuli, and CP subtypes used to address attentional biases and related processes. However, variation across studies did not allow for a decisive conclusion about the role of stimulus, task type, or related attentional processes. In addition, a table of CP attention-related models was produced and tested for reliability. Finally, other results and recommendations are discussed.

## Abstract 2

Supplemental Digital Content is Available in the Text.

## 1. Background

Chronic pain (CP) is characterised by distress and unpleasant sensations that last beyond 3 months.^52^ Chronic pain has a high prevalence worldwide, with around 20% of the world's population suffering from CP at some point in their life.^58^ Because of its nature, CP can lead to secondary complications, including attentional dysfunction.^2^ Difficulties in emotion processing add to the development and maintenance of CP.^23,49^ Theories concentrating on attentional processing have suggested that dysfunctional attention toward emotional information (ie, attentional biases towards negative emotions) might be one potential developmental and maintenance factor of CP.^36^ Cognitive biases such as attention to interpretation and recall of pain can lead to maladaptive strategies and the exacerbation of pain.^76^

Different developments and technologies have been used in the selective attention (SA) pain field since the first related experiment by Pearce and Morley.^68^ The use of the attentional bias concept became more common throughout the literature on SA in CP individuals after being adopted from earlier literature about biases in the context of anxiety.^5,51^ Attentional bias towards pain-related information is believed to be crucial in developing and maintaining the fear of pain in individuals with CP.^83,89^ Different mechanisms are believed to be involved in CP symptom maintenance.^28,90^ The key processes and characteristics that may be involved (illustrated in Table 1) include *hypervigilance*, in which the individual has a high level of alertness toward environmental triggers. Although still underspecified as a process (and sometimes used as an alternative general term for SA), hypervigilance contains the component of the “predisposition” to search for information actively and, thus, differs from the general concept of SA.^3,20,73^ The other somewhat similar process is *facilitated attention*, which means a faster orienting of attention towards a stimulus compared with another when it appears and being grabbed by it, especially for people of more “attention sensitive” nature.^18^ The *difficulty of disengagement* means being slower to disengage from a threatening stimulus and attend towards another stimulus. Other studies differentiate this process from *avoidance,* which is faster disengagement from threatening information (see also Brown et al).^11,83^ An important distinction is that attentional deployment can be s*trategic* that takes longer, like deliberately paying attention to where you expect someone to appear or *automatic*— the quick and unintentional processing of information.^79^ However, there is still some argument about the particular mechanisms and the role of earlier engagement or differences in disengagement in developing attentional bias and whether it is strategic or automatic.^54,78^ For example, if people with CP aim to control their pain, then pain-related cues become goal relevant for them. Therefore, they are deliberately (ie, strategically) allocating more resources to them.^85^

**Table 1:** Attentional processes measured by attentional bias tasks in main chronic pain interpretative models and hypotheses.

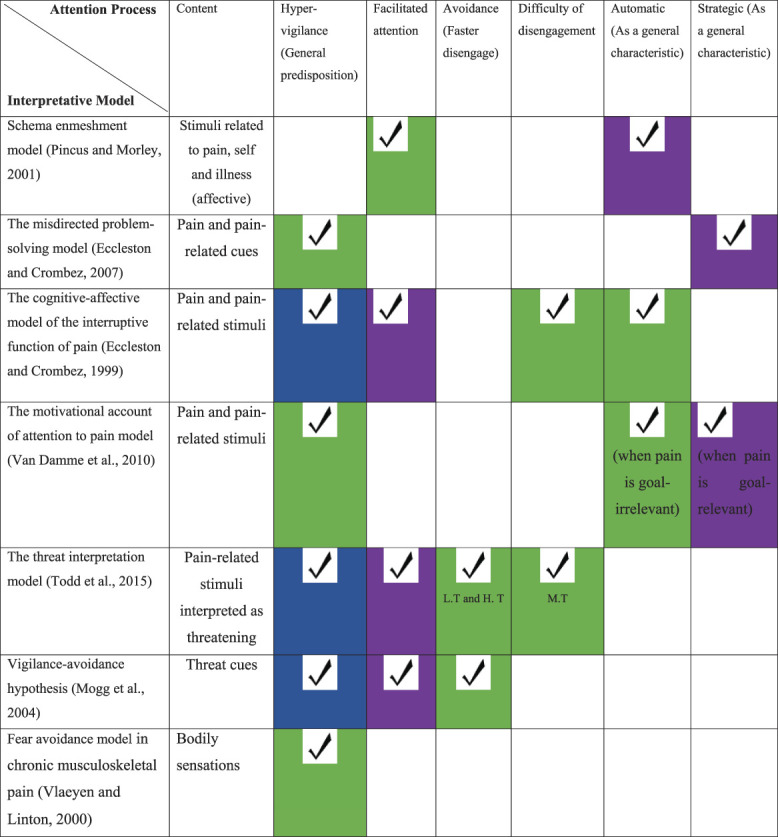

Interrater agreement is coded using colours where green: agreement between Ahmad Abudoush (AA) and Ellen Poliakoff (EP), blue: AA only, and purple: EP only.

H.T, high threat; L.T, low threat; M.T, moderate threat.

All of these processes have been introduced in the literature based on evolution in theoretical models in the field of pain. The main interpretative models were used to explore attentional processes in individuals with CP (Table 1). They try to describe what happens when individuals with CP are exposed to pain-related stimuli. The aim of producing a table with attention-CP related models is not to argue that the role of attention is distinctly different in the different models but rather to closely examine the dominant models and depict how attention is characterised in them. This is especially important since the tasks chosen for testing attentional biases are an essential criterion which determine the processes involved. For instance, hypervigilance or facilitated attention could be followed by avoidance (faster disengagement) or slower disengagement.^60,83^ Specifically, these models propose theoretical accounts of how the relationship between CP and attention might work, in which, attention might lead to greater awareness or processing of pain-related stimuli. The interpretative models (Table 1) agree that biases in attention to painful or pain-related stimuli in people with CP are key in the development and maintenance of CP or related disability. Yet, although all these models point at the role of attention, only a few specify the attentional processes they predict to be involved in biased attention for pain information. Furthermore, there are overlapping processes between some models, which differ from the interrelated and interacting varied cognitive biases.^87^ Because the exact processes are often left unspecified, in the current study, we aimed to indicate which processes are suggested to play a role and how they differ according to different models. It is worth emphasising that the attention part often forms only part of these models. In addition, it is worth noting that different attentional processes might be relevant at different time points in the development and maintenance of CP.^83^ To help understand, we coded the attentional processes specified by the different interpretative models (Table 1).

Following these theories, research in the domain has grown exponentially. Yet, research investigating attentional biases toward pain-related information in individuals with CP shows mainly either small to moderate effects, focusing on specific tasks, or having mixed findings.^1,21,46,78,84^ Although most of these meta-analyses outlined the nature of some attentional processes, they did not particularly emphasise the different processes and how related models interpret them. Furthermore, there is no gold standard task to assess attentional processing in this population or to produce reliable individual differences.^42^ Available meta-analyses have shown the existence of attention bias in CP individuals while processing pain-related information.^21,78^ However, one potential source of heterogeneity relates to the small sample size in some studies included in these meta-analyses. Thus, conducting a meta-analysis that includes studies with relatively large sample sizes would give a reliable and clearer image of the processes involved. Another potential source of heterogeneity relates to the paradigm used to research attention bias. The most commonly used task to assess attentional bias in CP populations is the dot-probe task.^84^ The dot-probe task assesses attentional biases by presenting a pain-related cue and a neutral cue simultaneously in 2 different locations followed by a target (ie, dot-probe) that appears in the place of either the pain-related cue (congruent) or the neutral cue (incongruent).^4^ The usual measure of attentional bias includes a combination of facilitated attending towards (faster responses to the probe that replaces the pain-related cue) and difficulty in disengagement (higher response latency to the probe when it replaces the neutral stimuli) from pain-related information.^18^ In the emotional Stroop task, both automatic and strategic SA characteristic processes are assessed, in which, attention is drawn to the meaning of the word (eg, sensory, affective, and neutral), and the participant must strategically attempt to ignore the meaning, which slows down the reaction time.^79^ These processes are assessed through measuring the differences in reaction time needed to name colours of words with neutral or pain-related semantics printed in different colours.^18^ Some researchers moved to use pictorial stimuli rather than words to access related affective information^50^ and to overcome cross-language barriers to help pooling data from different studies on the long term. However, the cross-cultural barriers cannot be simply removed by the mere use of images because differences could be attributed to the differences in the cognitive cultural schemas of images meaning.^8,47^ Also, different meta-analyses found that using words produces large effect size compared with pictorial stimuli.^21,61^ For the dot-probe task, some studies have reported inconsistent findings and replication and validity difficulties.^25,82^ Furthermore, the dot-probe task does not usually include a baseline measurement. This means that it can be challenging to measure the facilitation of attention or to separate it from disengagement without combining it with other procedures, such as having a neutral–neutral stimulus as a baseline measurement (see Fashler and Katz,^32^ but also Blicher et al.^9^ who questioned the use of neutral-neutral trials), or adding an eye-tracking task.^18^ The study by Fashler and Katz^32^ is an example of including the measurement of a neutral–neutral condition. Sometimes, manipulation of the stimulus onset asynchrony (SOA) is used to look at the automatic and strategic elements of attention.^27^ Different tasks have advantages and disadvantages. For instance, the dot-probe task measures the competition between stimuli, however, as discussed above this can lead to difficulties with interpretations of the results. Meanwhile, the Posner cueing task does not measure competition but can be used to look at the time course of attention and can be set up to examine strategic or automatic attention.^18^ It uses covert orienting of attention, in which an individual is paying attention without moving their eyes. Reaction times are measured toward different cues, which are presented singly.^45^ In the Posner cueing task, pain-related cues are either valid (ie, congruent location of the pain-related cue and the target) or invalid (ie, incongruent location of the pain-related cue and the target), in which the cue is presented first, followed by the target. This allows the separation between the processing of different cue types.^18,41,77^ However, it is worth mentioning that because Stroop and Posner cueing tasks present only one cue in each trial, the measurement can include response bias in addition to the attentional bias. This limitation can be resolved by including a discrimination task, in which, the task would include distinguishing the target type (eg, colour degree) rather than determining only its spatial location. Furthermore, this limitation was one of the factors that led to the development of the dot-probe task.^53^ A brief description of the main reaction time tasks can be found in Table 2.

**Table 2:** A brief description of the main reaction time tasks used in assessing attentional biases in individuals with chronic pain.

Task	Brief description
Modified Posner (spatial)-cueing task	An emotion-related or neutral cue is presented at one of 2 possible locations in the visual periphery before a neutral target appears. The participant is then asked to identify the target type or spatial location as quickly as possible. The cue may be predictive (indicate the likely location of the upcoming stimulus) or nonpredictive (location unrelated to the target location)
Emotional Stroop task	An emotion-related or neutral coloured word (stimulus) is presented against a grey background. The participant is asked to identify the colour of the word regardless of the word's semantic meaning as quickly as possible, and reaction time is recorded
Dot-probe task	Two cues (one neutral and one emotion-related) are presented at opposite locations in the visual periphery and then a target appears. The participant is asked to identify the target type as quickly as possible. Differences in reaction time between congruent trials, where the dot replaces the emotion-related stimulus and incongruent trials, where the dot replaces the neutral stimulus are recorded
Visual search task	The individual is asked to find a particular visual stimulus (ie, target) among distractors (ie, cues) as quickly as possible. Two conditions are usually presented; in the first one, the distractors are pain-related, and the target is neutral, and in the second, the distractors are neutral, and the target is pain-related. Reaction time for both conditions is recorded

Recent reviews in the CP field have focused on only one specific task, such as the dot-probe task,^84^ eye-tracking,^46^ or emotional Stroop task,^1^ or one specific CP subtype.^66^ Moreover, for reviews that did explore different tasks, the lack of consistency between experimental results is still one of the most common barriers toward understanding the SA processes in this population.^21^ However, since the publication of this review, many novel studies have been published in this domain. Therefore, understanding the similarities and differences between SA tasks and their interpretative theoretical models is crucial.^18,55^ Another major pitfall noticed in previous studies was the heterogeneity of CP samples. The nature of the SA processes affected could differ between subtypes of CP.^22,38^

We conducted a systematic review and a meta-analysis which aimed to examine whether (1) different experimental tasks identify differences in SA processes between individuals with CP and healthy controls; (2) different tasks and stimuli are more likely to detect differences in SA processes between CP individuals and healthy controls; (3) patterns of differences in SA processes differ across subgroups of individuals with CP (eg, chronic low back pain [CLBP], fibromyalgia, complex regional pain syndrome [CRPS], and headache).

## 2. Methods

The systematic review protocol was prepared and registered on PROSPERO (CRD42019159121), and the systematic review was conducted and reported according to the Preferred Reporting Items for Systematic Reviews and Meta-Analyses (PRISMA) 2020 guidance.^67^

### 2.1. Searches

Four databases were searched from inception to August 17, 2022, including the Ovid platform (title and abstract), MEDLINE, PsycINFO, and PsycARTICLES full text (by Ovid), and the Web of Science. The searching strategy is included in Appendix A (available as supplemental digital content at http://links.lww.com/PAIN/B793). Furthermore, the Open Grey database was searched for relevant grey literature. Medical subheading heading (MeSH) terms were used where available (ie, Medline and PsycINFO databases) using chronic pain as a subheading. Journal articles and PhD theses were included but not conference abstracts and MSc theses. Backward citation tracking was conducted by searching reference lists of included studies. Related systematic reviews were hand searched with forward citation tracking involving searching the references of any relevant systematic reviews.

### 2.2. Study selection

The study selection was completed in 2 stages. First, we started with titles and abstracts screening, followed by full-text screening for eligible studies at the title or abstract screening stage. The 2 stages of screening were performed independently by 2 researchers (A. N. Abudoush and A. Noureen). The interrater agreement (*κ*) on searching the titles and abstracts and full-text screening reached 97.38% and 85.83%, respectively.

### 2.3. Eligibility criteria

We had the following eligibility criteria using the Population, Intervention, Comparison, Outcomes and Study (PICOS) framework:

(1) Population: CP group: individuals with CP for 3 months or more, aged 18 years or older, and have normal or corrected to normal vision.
Control group: healthy individuals without CP, aged 18 years or older, and have normal or corrected to normal vision.
(2) Intervention: these included experimental conditions such as (a) tasks that assess attention bias for pain-related information or (b) any experimental pain-related stimulation such as auditory, visual, or somatosensory.
(3) Comparison: baseline, neutral condition, or no comparison.
(4) Outcome measures: reaction times were the primary outcome measure used to compare tasks and stimuli used. Attentional biases of participants with (ie, at least 50% of the sample size) and without CP were compared. This was performed by comparing the reaction time differences between participants with and without CP. Between-group differences and within-group differences were calculated. Some studies used an equation to calculate the bias score (ie, attentional bias index) instead of the reaction time. The bias score relied on the differences between the CP group and the control group (some other studies that only included one group were excluded only from the meta-analysis part). The magnitude and orientation of these scores were used to infer the SA process that might occur.
(5) Study design: for the systematic review, we included studies with experimental or quasi-experimental designs using any reaction time-based experimental task that assessed attentional biases and had at least 20 participants. For the meta-analysis, a further condition of having a control group with at least 20 participants for comparison purposes.

We excluded studies that were nonexperimental (eg, qualitative studies), used questionnaires measures only (eg, cross-sectional using questionnaires only), were not focused on chronic pain, or were focused on drugs (eg, pain killers and opioids). Because previous meta-analyses reported a high level of heterogeneity,^21,84^ we excluded studies that had less than 40 participants (ie, at least 20 participants in each arm) to improve the reliability of this review. Thus, based on the inclusion criteria, nonreaction time-based eye-tracking studies were excluded from this review. Such eye-tracking studies were explored in a recent meta-analysis.^46^ Other exclusion criteria were tasks not including attention bias toward pain-related stimuli and studies not written in English.

### 2.4. Data extraction

Two researchers (A. N. Abudoush and A. Noureen) completed the data extractions independently using a standardized excel spreadsheet piloted before its use. We extracted information on studies, populations, experimental tasks, comparisons and outcomes, and quantitative data that were amenable for the meta-analysis (Appendix C, available as supplemental digital content at http://links.lww.com/PAIN/B793). Demographic information (ie, age, gender, ethnicity, education, and socioeconomic status) of the participants, pain characteristics (ie, duration, intensity, and location[s]), and measurement tools used in the studies were summarised in Appendix D (available as supplemental digital content at http://links.lww.com/PAIN/B793). When these values were not presented separately in the journal articles, we gathered them through contacting the authors of these articles or authors of previous meta-analyses. If the experimental study had more than 2 groups, we chose the group with the least comorbid symptoms to increase the homogeneity of the overall sample.^6,35,39,62^ When there was a discrepancy between the 2 researchers involved in the data extraction, it was resolved by discussions or the involvement of the wider team of reviewers.

### 2.5. Quality assessment

We assessed the included studies' quality using a tool adapted from Crombez et al.,^21^ which involved assessing the internal and external validity of the eligible studies. Although we used the tool as it is, we changed the scoring system to better interpret the overall quality of scoring (Appendix B, available as supplemental digital content at http://links.lww.com/PAIN/B793). The score on each item ranged from 0 to 3 (expect one item ranging from 0 to 4); then, this score was converted to a percentage ratio for a more straightforward interpretation. Two researchers (A. N. Abudoush and A. Noureen) completed the quality assessment independently, and disagreements were resolved by discussion.

### 2.6. Data analysis

The results were initially narratively synthesised according to the research aims with a focus on different tasks, stimulus types, and CP subgroups. For the subset of studies with amenable quantitative data, meta-analysis was conducted using Stata-16 software.^44^ Hedges' g was used to calculate the effect size because it is better with smaller sample sizes compared with Cohen d. The values of Hedges' g can be interpreted similar to those of Cohen d, which range between 0.01, 0.2, 0.5, 0.8, 1.2, and 2.0, indicating very small, small, medium, large, very large, and huge effect size, respectively.^13,19^ When quantitative data were not available, we twice contacted the authors of the eligible studies included. Subgroup analyses were executed to examine the effect of different tasks, stimuli, and CP subtypes. A sensitivity analysis was performed to examine whether effects remain robust when only studies with low risk of bias scores are retained in the analyses (Appendix E, available as supplemental digital content at http://links.lww.com/PAIN/B793). All analyses were conducted using the DerSimonian–Laird random-effects model to account for between-study heterogeneity. Heterogeneity was quantified using the I^2^ statistic. Conventionally, I^2^ values of 25%, 50%, and 75% indicate low, moderate, and high heterogeneity.^43^ Provided we identified more than 10 studies per outcome,^80^ a formal assessment of slight study bias (publication bias) was performed by constructing funnel plots (with the use of metafunnel command) and examining the value and significance of the Egger test (using the metabias command).^31,40,81^

## 3. Results

### 3.1. Studies selection

**Figure 1.**
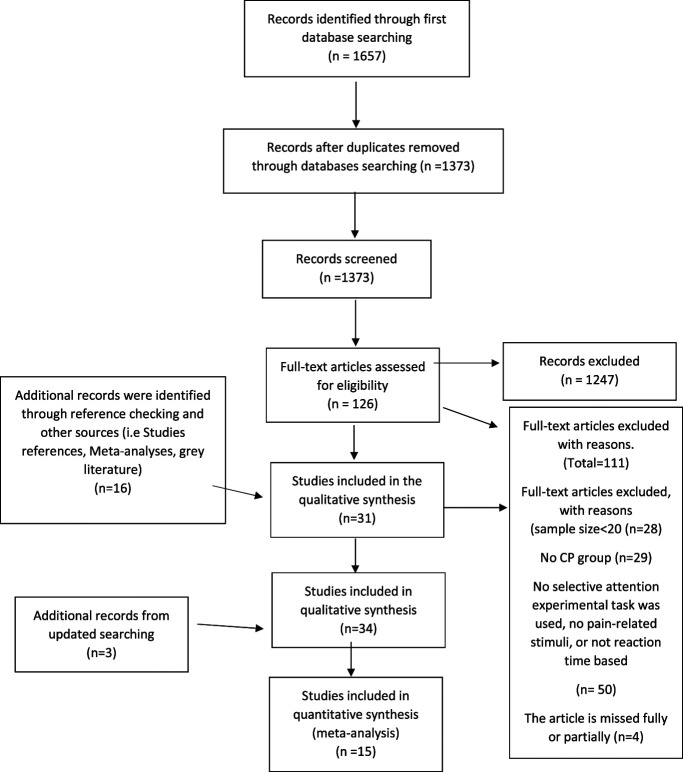
Preferred Reporting Items for Systematic Reviews and Meta-Analyses (PRISMA) flow diagram that illustrates the current progress in the systematic review. CP, chronic pain.^63^

The systematic searches revealed 1657 studies. After removing duplicates (284 studies), 1373 studies from 4 bibliographic databases and 2 studies from grey literature were retained for titles and abstract screening. Of these studies, we identified 126 studies for full-text screening. Another 2 search updates revealed 361, 143, and 131 studies, respectively, totalling 2008 studies for screening. A total of 34 studies were included in the review, and of these, 15 studies were included in the meta-analysis. The details of the systematic review process are illustrated in Figure 1 using the PRISMA flowchart.

### 3.2. Descriptive characteristics of studies and outcomes

The overall sample size of 3154 individuals across the studies included 2057 CP individuals and 1097 healthy participants. The sample size for CP groups ranged from 20 to 170 and from 20 to 200 for the control groups. The mean age of the samples for CP groups (M = 42.61, SD = 7.19) and healthy groups (M = 33.85, SD = 10.35). The samples predominately consisted of females, mean ratio of females (65.0%) in CP groups and (64.6%) in control groups. Most studies were conducted in high-income countries (29 of 34 studies, 85.3%). The average pain duration was 95.02 months (SD = 55.22; range, 6.7-220.32), and the average pain intensity (ie, of 10°) was 4.62 (SD = 1.27; range, 2.5-6.4) (see Appendix D for the pain characteristics and Appendix F for the included studies' full list, available as supplemental digital content at http://links.lww.com/PAIN/B793). Most experiments k = 21 were conducted in the English language. The first study was published in 1998 by Pincus et al.,^70^ and the last was published in 2020 by Carleton et al.^16^ The descriptive characteristics of all studies included in the systematic review are summarised in Table 3.

**Table 3:** The descriptive characteristics of all studies included in the systematic review.

First author	Study setting (country)	Research design	Pain intensity M (SD)	CP subtype	Procedure language	Comorbid symptoms of EG	Sample size (EG)	Sample size (CG)
*Andersson and Haldrup (2003)	Sweden	Quasiexperimental/2 matched groups	NPRS 0-10 6.25 (1.3)	FM, whiplash, and LBP	Swedish	Anxiety and depression	20	20
*Asmundson et al. (2005)	Canada	Quasiexperimental/2 groups design	MPI-pain subscale 4.1 (1.2)	83% BP	English	Anxiety and depression	36	29
Asmundson and Hadjistavropoulos (2006)	Canada	Quasiexperimental/2 matched groups (secondary reanalysis)	—	83% (n = 30) BP	English	Anxiety, FOP, and depression	36	29
*Beck et al. (2001)	USA	Quasiexperimental/3 groups design	PTSD with CP 4.33(1.17) CP 3.80 (1.44)	Musculoskeletal	English	PTSD and other nonspecified psychiatric diagnoses	26	21
*Carleton et al. (2020)	Canada	Quasiexperimental/2 groups design	—	FM	English	Anxiety, depression, and stress	26	29
Chapman and Martin (2011)	UK	Quasiexperimental/2 groups design	51.35 (19.21)	IBS	English	Anxiety and depression	20	33
Crombez et al. (2000)	Belgium	Quasiexperimental/one group design	48.72 (24.88)	LPB	Dutch	Anxiety	25	NA
Dear et al.^26^ (2011)	Australia	Quasiexperimental/2 groups design	Not specified	44% LBP and limbs; 17% upper shoulder and cervical region	English	—	139	200
*Dear et al.^25^ (2011)	Australia	Quasiexperimental/2 groups design	Not specified	LBP 43%; upper shoulder and cervical 17%	English	Depression, stress, and anxiety	129	50
Dehghani et al. (2003)	Australia	Quasiexperimental/one group design	MPI 4.07 (1.13)	LBP (37.5%), upper-limb pain (15%), lower-limb pain (14%), and 6% cervical pain	English	Moderate disability, depression, anxiety, FOP, and severe stress	168	NA
Dehghani et al. (2004)	Australia	Quasiexperimental/one group pretest–posttest design	MPI 4.25 (1)	CLBP (38.1%), upper limb pain (12%), lower limbs (12%), cervical pain (12%).	English	Moderate disability, FOM, and FOP Severe stress, clinical depression, and anxiety	42	NA
*Duschek et al. (2014)	Germany	Quasiexperimental/2 groups design	MPQ 53.8 (16.5)	FM	German Deutsch	Depression and anxiety	27	34
Fashler and Katz (2014)	Canada	Quasiexperimental/2 groups design	Mild (15.7%), moderate (74.5%), severe (9.8%)	Neck or BP (30), headache/migraine pain (22), ankle/knee (21), shoulder (15) stomach (12) hip (5), arm (2), eye (1), and jaw (1)	English	Severe disability and anxiety	51	62
*Fashler and Katz (2016)	Canada	Quasiexperimental/2 groups design	Mild (15.7%), moderate (74.5%), and severe (9.8%).	Neck and/or BP (30), headache/migraine pain (22), ankle/knee (21), shoulder (15) stomach (12) hip (5), arm (2), eye (1), and jaw (1)	English	Severe disability and anxiety	51	62
*Franklin et al. (2016)	UK	Quasiexperimental design/4 groups design (defensive high-anxious, high-anxious, repressor, non-extreme, and control)	—	CBP	English	Anxiety	70	20
Garland and Howard (2013)	USA	Preallocation assessment/2 groups RCT	MORE 5.82 ± 1.27 Support 5.94 ± 1.59	Lumbago (58%), FM (19%), arthritis (13%), and cervicalgia (3%)	English	MDD GAD Substance use PTSD	92	NA
*González et al. (2010)	Spain	Quasiexperimental/2 groups design	—	FM	Spanish	Anxiety	25	25
*Haggman et al. (2010)	Australia	Quasiexperimental/4 groups design (CLBP-PT/CLBP- tertiary referral pain clinic/acute LBP/control)	PT 2.61 (2.25) Tertiary 4.85 (2.81)	LBP	English	Depression	107	50
Harvold et al. (2018)	Denmark	Quasiexperimental/2 groups design	CP no PTSD 6.4 ± 1.6 CP and PTSD 7.4 ± 1.5	RTA related noncancer CNP	Danish	PTSD	20	NA (CP + PTSD group less than 20)
Jackson et al. (2019)	China	Quasiexperimental/one group sample design	CPGS 12.66 (4.82)	Neck or shoulder (46%) LBP (28%) Extremity (13%) Head or face (10%) Others (3%)	Chinese	—	89	NA
*Khatibi et al. (2008)	Iran	Quasiexperimental/2 groups design	VAS (100 mm) 54.6 (13.6)	Not specified	Persian	Anxiety, depression, stress, FOM, and FOP	170	40
Liossi et al. (2010)	UK	Quasiexperimental/2 groups design	2.5 (1.1)	CH (tension type)	English	Anger trait, angry temperament, angry reaction, and anger out	40	40
Mazidi et al. (2019)	Iran	Quasiexperimental/2 groups design	VAS current week 5.34 (2.24)/VAS currently 2.7 (2.17)	17.85% upper limbs, 21.43% lower limbs, and 10.71% BP	Persian	Anxiety, depression, and stress	28	29
Mohammadi et al. (2012)	Iran	Quasiexperimental/3 groups design (CP, caregivers, and control)	VAS—100 mm CP 51.41 (30.71) caregivers' estimation 53.71 (28.43)	20.7% upper limbs, 23.7% lower limbs, 43% BP, and 12.6% more than one location	Persian	Nonsig. on anxiety, depression or stress	135	52
Peters et al. (2000)	Netherland	Quasiexperimental/2 groups design	—	FM	Dutch	Nonspecific bodily symptoms	30	30
*Pincus et al. (1998) (experiment 1 only)	UK	Quasiexperimental/2 groups (2 × 4 factorial design)	NPRS scale 1-101 30 (17)	—	English	Anxiety and depression	20	20
*Roelofs et al. (2005)	Netherland	Quasiexperimental/2 groups design	Using VAS-100 mm 60.1 mm (26.3)	CLBP	Dutch	Substantial physical disability	49	44
Schoth and Liossi (2013)	UK	Quasiexperimental/2 groups design	3.11 (1.22)	CH	English	Depression and state and trait anxiety.	37	38
*Schoth et al. (2014)	UK	Quasiexperimental/2 groups design	—	Tension-type headache 19 (83%) Migraine 4 (17%)	English	Severe disability	23	24
Sharpe et al. (2009)	Australia	Quasiexperimental/one group design	5.6 (9.9)	Definite or classic rheumatoid arthritis	English	Anxiety	100	NA
Sharpe et al. (2012) (study 2 only)	Australia	Preallocation RCT (study 2 only)	ABM + CBT 4.45 ± 2.0 PT + CBT 3.3 ± 2.0	Benign CP Arthritis	English	Disability	34	NA
Snider et al. (2000)	Canada	Quasiexperimental/2 groups design	MPQ-SF 5.6 (2.1)	CBP (23) CNP (10)	English	Depression	33	33
Van Ryckeghem et al. (2012)	Belgium	Quasiexperimental/one group design	MPI scale 3.86 (SD = 0.98)	CBP (92.8%), CNP (68.1%) Leg pain (66.7%) Arm pain (44.9%)	Flemish	Depression, disability, and anxiety	69	NA
*Yang et al. (2013)	China	Quasiexperimental/2 groups sample design	Using CPGQ High FOP group 16.85 (4.06) Low FOP group 16.27 (1.90)	Abdominal pain (12) Headache (4) BP (3) Orofacial pain (2) Shoulder pain (1) NP (1) Chest pain (1)	Chinese	FOP	24	24

* Study included also in the meta-analysis.

ABM, attentional bias modification; BP, back pain; CBP, chronic back pain; CBT, Cognitive Behavioural Therapy; CH, chronic headache; CLBP, chronic low back pain; CP, chronic pain; CPGQ, Chronic Pain Grade Questionnaire; DP, dot-probe task; FM, fibromyalgia; FOM, fear of movement; FOP, fear of pain; FOP, fear of pain; FV, free viewing task; GAD, generalised anxiety disorder; LBP, low back pain; MDD, major depressive disorder; NP, chronic neck pain; NPRS, Numeric Pain Rating Scale; PC, Posner cueing task; PTSD, posttraumatic stress disorder; RTA, road traffic accident; S, Stroop task; VAS, pain severity: Visual Analogue Scale.

Performance related to attentional biases of participants with CP was compared with healthy participants (25 studies of 34 have a control group, 73.5%). This was conducted by comparing the reaction times toward sensory pain-related information and affective pain-related information with reaction times toward neutral information. When we had mixed types of pain-related stimuli in the same study, we explored each of them separately. Across the studies, different SA processes were implicated by the authors. Although most studies that used the dot-probe task predicted hypervigilance process, they linked this process to a range of models, mainly the fear-avoidance model,^89^ fear of reinjury model,^88^ and the vigilance-avoidance hypothesis.^60^ On the other hand, not all studies linked a particular model to the same attentional processes; instead, different findings were occasionally used to support the same model depending on how they were interpreted. The fear-avoidance model was linked with combinations of hypervigilance, avoidance, and difficulty of disengagement processes when using the dot-probe task (see Table 3-A in Appendix C, available as supplemental digital content at http://links.lww.com/PAIN/B793).^89^ For instance, some studies using eye-based reaction times found that CP individuals tend to be hypervigilant to, and then avoidant from painful facial expressions, which supports the vigilance-avoidance hypothesis.^32,57,91^ The main task characteristics of all studies, including the attentional processes, are listed in Table 3-A, Table-3-B, and Table 3-C in Appendix C (available as supplemental digital content at http://links.lww.com/PAIN/B793).

### 3.3. Risk of bias assessment

Internal and external validity was assessed using the tool adapted from Crombez et al.^21^ meta-analysis; the fulfilment of the internal validity between studies reached (79.3%), and the external validity fulfilment reached (78.7%). Detailed information on the quality ratings can be found in Appendix B (available as supplemental digital content at http://links.lww.com/PAIN/B793).

### 3.4. Synthesis of results

The studies included in the analysis varied in the experimental tasks and type of stimulus used. Therefore, we grouped the results for each main task and stimulus type used. For the CP subgroups, we compared studies related to back pain only due to the heterogeneity of other study samples.

#### 3.4.1. Tasks

The main tasks used to assess CP are the modified Stroop and dot-probe tasks, either with or without eye-tracking (ie, eye-tracking technology was used in k = 6 studies). For the type of tasks used, the main tasks were the modified dot-probe task k = 23, which used pain-related stimuli (ie, pictorial or words) and the modified Stroop task k = 8, which used words as pain-related information, with one study (Asmundson et al., 2005) using both tasks consecutively. Other tasks included the Posner spatial cueing task k = 2, dual-task (ie, detection of electrical stimulation task and reaction time [RT] for visual stimuli task) k = 1, and visual search task k = 1.

#### 3.4.2. Stimulus

For the stimulus type, words were used in k = 25 studies, whereas pictorial stimuli were used in k = 11 studies, while 2 studies used both words and pictorial stimuli in their study separately.^26,75^ Other stimuli included using geometric pictorial shapes k = 2; first study used a red light as a signal before detecting the location of an innocuous electrical stimulus (ie, bodily sensation relevant to pain) and determining a geometric object type in a dual task experiment.^69^ The second study used a pink or blue square and a noxious electrical stimulus (one of the squares was related to pain through classical conditioning).^86^ The results of extraction are shown in Appendix C—Table 3 (available as supplemental digital content at http://links.lww.com/PAIN/B793).

#### 3.4.3. Chronic pain subtypes

Regarding the CP subtypes, the results were heterogeneous. The studies were connected to the CP subgroup category with the main diagnosis (ie, 50% or more of the sample size); back pain k = 11, fibromyalgia k = 4 chronic headache k = 3, irritable bowel syndrome (IBS) k =1, rheumatoid arthritis (RA) k = 1, and road traffic accident (RTA) k =1. In comparison, the rest of the studies (ie, k = 11) recruited samples with miscellaneous subtypes of CP and not specified in further one study k = 1. However, only a few studies were amenable for inclusion in the meta-analysis (ie, chronic back pain k = 4).

#### 3.4.4. Attentional characteristics and processes

Different processes were identified to be involved in different phases of the attentional process as stated in studies included. Although hypervigilance k = 20, avoidance k = 5, and facilitated attention k = 1 attention processes were linked to the initial phase of the attentional process (ie, <200-300 ms), the difficulty of disengagement process k = 8 and strategic characteristics were related to later processing (ie, >200-300 ms).^7,33,65^ Although the strategic characteristic was reported in k = 9 studies, automaticity was not explicitly discussed but was implied through descriptions of facilitated attention. All attentional processes and their related models are summarised in Table 1. Ratings in Table 1 were performed by 2 independent reviewers (A. N. Abudoush and E. Poliakoff), and the interrater reliability was medium (kappa coefficient = 0.53).

For the presentation time of the stimulus, many studies, k = 14, used 500 milliseconds. Although some studies used shorter presentation time (ie, 100-300 ms) k = 3, others used longer presentation time (1000 ms to 4000 ms) k = 9, and some studies k = 4 used both short and long presentation time. Also, in the Stroop task some studies used unlimited presentation time k = 5, with 2 of them contained also masked presentation of 14.3 milliseconds that then replaced by a string.

#### 3.4.5. Main comorbid symptoms

Regarding comorbid psychological symptoms, depression and/or anxiety were the main comorbid symptoms of individuals with CP k = 24, posttraumatic stress disorder k = 2, anger trait k = 1, miscellaneous sample symptoms k = 2, unspecified symptoms k = 2, in addition to general comorbid disability k = 3. Where possible, the results of the studies not included in the meta-analysis, including subgroup analysis, were described in Appendix C—Table 3 (available as supplemental digital content at http://links.lww.com/PAIN/B793).

### 3.5. Meta-analysis

**Figure 2.**
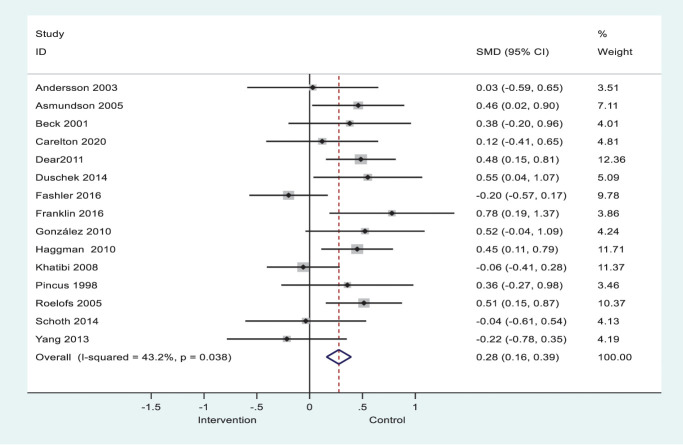
Forest plot of the effect sizes for the sensory pain–related information for all studies.

**Figure 3.**
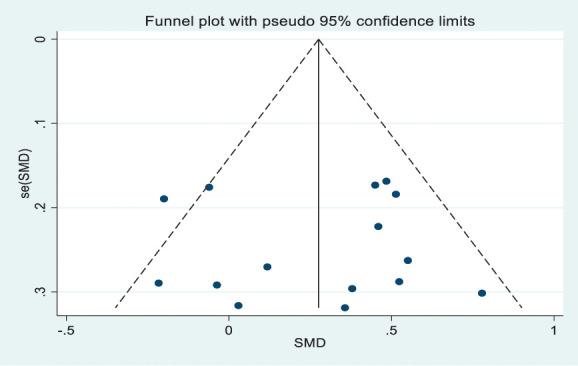
Funnel plot for the assessment of symmetry and publication bias for all studies.

The pooled effect size across the 15 studies was small and significant (g = 0.28, 95% CI [0.16, 0.39], I^2^ = 43.2%, *P* = 0.038); see forest plot in Figure 2, suggesting that CP groups have a greater bias towards sensory pain-related information than healthy groups. As indicated by the I^2^ statistic, there was moderate heterogeneity in this meta-analysis. However, we found no evidence of publication bias (*P* = 0.98, 95% CI [−3.26, 3.18]), as shown in Figure 3. Sensitivity analysis was conducted using the highest 10 studies' quality score, and the effect size was moderate and significant (g = 0.39 95% CI [0.24, 0.54], I^2^ = 15.4%, *P* = 0.301). This supports the primary results that the CP population has a greater attentional bias toward sensory pain-related information. For assessing the effect size of the affective pain-related information, data from authors were obtained from only 3 studies, which were found to be moderately significant (g = 0.48, 95% CI [0.23, 0.72], I^2^ = 7.1%, *P* = 0.341) (Appendix E, available as supplemental digital content at http://links.lww.com/PAIN/B793).

#### 3.5.1. Effect of task

For the task comparison, 6 studies used the dot-probe task, and the revealed pooled effect size was small and significant (g = 0.22, 95% CI [0.06, 0.38], I^2^ = 67.4%, *P* = 0.009), whereas the pooled effect size for 6 studies that used the Stroop task was moderate (g = 0.41, 95% CI [0.18, 06], I^2^ = 0%, *P* = 0.853) (Appendix E, available as supplemental digital content at http://links.lww.com/PAIN/B793).

#### 3.5.2. Effect of stimulus type

Regarding the type of stimulus, the pooled effect size across 10 studies that used words was moderate and significant (g = 0.44, 95% [0.28, 0.60], I^2^ = 0%, *P* = 0.681), whereas the pooled effect size across 5 studies that used a pictorial stimulus was small and nonsignificant (g = 0.09, 95% [−0.10, 0.28], I^2^ = 61.3%, *P* = 0.035) (Appendix E, available as supplemental digital content at http://links.lww.com/PAIN/B793).

#### 3.5.3. Effect of chronic pain subtypes

For the CP subgroups, we found a significant and moderate effect size (g = 0.51, 95% [0.31, 0.71], I^2^ = 0%, *P* = 0.810) (Appendix E, available as supplemental digital content at http://links.lww.com/PAIN/B793), related to chronic low back pain in 4 studies. Other CP subtypes could not be analysed using meta-analysis due to heterogeneity and lack of studies focused on CP subgroups.

## 4. Discussion

In this systematic review, we explored reaction time-based studies and synthesised a descriptive analysis that shows overlap and variation between tasks, stimuli, CP subgroups, attentional processes predicted, and interpretational models used in these studies. For the meta-analysis, we found that using relatively larger sample size confirmed that compared with healthy controls, individuals with CP show an attention bias towards sensory pain information of relatively small to moderate effect size. Furthermore, different from previous reviews that looked within groups (ie, compared congruent and incongruent within groups), we found a moderate effect size for affective pain-related information when assessing studies with large sample size. However, only limited affective pain-related data (ie, k = 3) were amenable for the meta-analysis, and therefore, we recommend caution in interpreting these findings. The low number of studies included is mostly attributed to either low sample size or poor data reporting. Thus, there is a need for more studies with robust methodology to be conducted using affective pain-related cues. We also found a moderate heterogeneity level, which is expected given the variation in methodologies and outcomes of the studies. Therefore, we followed this up with subgroup and sensitivity analyses to explore key sources of the detected heterogeneity. The main tasks used were the dot-probe task and the emotional Stroop task. The emotional Stroop task identified greater attentional biases in the CP groups compared with controls. However, this might be due to the involvement of different attentional processes in the tasks, or even other nonattentional processes, such as higher-order processes, when the tasks are not purely measures of attentional biases. Therefore, further studies are needed to confirm such differences. The main stimulus types were words and pictorial stimuli; however, we could not determine whether there was greater variability in the type of images used than the words used because many studies did not include the actual stimuli list used in the experiment, and thus, a transparent reporting of stimuli is needed.

Our findings concur with previous reviews, which also observed high inconsistency across studies.^21,84^ In comparison with other recent reviews which investigated only dot-probe task,^84^ eye-tracking studies,^46^ or pain-related and bodily somatosensory stimuli,^10,21^ this review explored all existing reaction time-based tasks. It should be noted, however, that our strict inclusion criteria meant that some of these reviews included more studies. This exhaustive review of assessment tasks and stimuli provides an overview of SA processes in the CP population and the associated attention processes (See Table 1). For instance, our review has found that some previous studies claimed that individuals with CP show *strategic* attention toward pain-related stimuli rather than *automatic* attention.^57,79^ This claim was consistent across different experimental tasks (ie, Stroop, dot probe, and eye tracking). Most studies that looked at automatic vs strategic processes chose a similar time point (ie, 500 ms) in the dot-probe task to assess the strategic or hypervigilance attention processes. Interestingly, it was noticed that this choice was mainly based on earlier research that found significant results for strategic but not automatic attention.^71,79^ Choosing such a time point implied that attention in the CP population is widely accepted as more related to strategic than automatic processes. However, having an agreed format to facilitate comparability of presentation times among studies from different countries one would need to run a more global study that takes into consideration the cross-cultural differences. Such cultural variations could be related either to the cultural sensitivity (eg, type of stimulus and the appropriateness of the stimulus) or language specificity of that culture (eg, language comprehension, language processing, language expression speed, and stimulus interpretation).^56,72^ Furthermore, this review indicates that studies using words more consistently found differences than those using pictorial stimuli (Appendix C, available as supplemental digital content at http://links.lww.com/PAIN/B793). Although words might be less effective at eliciting memories or schemata, they have been used more often in studies of CP. Furthermore, another novel feature of this systematic review is that it explored the general SA processes in light of their relation to the different tasks and the interpretative models (Table 1). By doing so, we tried to link SA processes with the theoretical models to understand how attention is characterised in them, taking into consideration the psychometric parameters from the different tasks, and stimuli.

We found from the descriptive analysis that the same task was often used to support different models. This is unsurprising given that there is an overlap in the SA processes implicated by the different models. This process–model relation could be a priori specified through preregistration in future studies, as recommended by the literature on the open science.^59^ Thus, the key importance of preregistration is that it prevents HARKing (ie, hypothesising after results known).^48^ Specifying the exact effects predicted in advance of collecting data through preregistration of the experiment is crucial for having consistency between predictions and the arguments made. Thus, effects might be overestimated without such preregistration.

This review has several research implications. First, we found a larger effect when using the Stroop task over the dot-probe task in measuring the bias toward (or away from) pain-related information, suggesting that bias toward pain-related information is greater when using the Stroop task rather than the dot-probe task. However, we need to be careful when interpreting these results because it is difficult to determine the exact driving factors because of the relatively small number of studies in subgroups with potential overlap between moderators, such as the type of task and the type of stimuli. Furthermore, because the effect is smaller it does not mean that the task is less good in detecting the attentional bias and related processes. The larger effect may be due to other processes (eg, higher-order processes) that have an effect. While a general argument about attentional bias can be made that it does not matter which task we are using because we should have an attentional bias effect. On the other hand, it is worth noting that the Stroop task calls on many attentional processes (such as automatic and strategic processes characteristics as well as inhibitory mechanisms),^12^ and the effect may be larger because they are added together, so it is hard to tease them apart. We recommend that future research use more than one task, which would allow for more robust results, such as the study by Asmundson et al.,^3^ use the task that is more reliable to detect the hypothesised SA process, or use repeated measurements. Furthermore, in this study, the authors correlated findings between the tasks and found that only affect pain-related and health catastrophe words were significant. These findings raise questions about whether these tasks are distinguishable from each other, given that they were correlated. Because only a few studies investigated other tasks and due to data reporting problems, these were not included in the meta-analysis. However, using other tasks such as the Posner cueing task might allow future researchers to focus on specific attentional subprocesses.^86^ Furthermore, few studies used actual bodily or painful stimuli, such as the study by Bulcke et al.^14^ that discuss hypervigilance for somatosensory signals in CLBP individuals. This could be a future direction compared with most studies that mainly used words or images.

Second, as expected, we found that many studies used the dot-probe task, which has also been used in studies investigating attentional bias modification as a potential intervention for some subtypes of individuals with CP.^15^ However, the psychometric properties of dot-probe tasks, such as reliability, have been questioned, and alternative tasks such as using the Posner cueing-target task have been recommended, which have more reliable psychometric properties, especially when controlling for *across* and *within* trial parameters.^41^ However, the Posner-cueing task has only been used in a small number of studies. It should also be noted that the limitations of the chosen task affect what attentional processes can be specifically measured and therefore used in interpretations. Also, recent developments in this field suggest using technologies such as eye-tracking—which given the continuous measurement—has pros and cons compared with relying on the reaction time. However, eye tracking cannot be used to measure attention to stimuli in the periphery,^57^ which is usually involved in capturing exogenous (external) stimuli.^7^ Furthermore, we cannot directly measure processes through eye tracking (ie, a person can pay attention to a location without moving eyes), so they are not inherently better than other tasks. Further information about the advantages and disadvantages of the eye-tracking technology were discussed in the study by Chan et al.^17^ It was obvious that many studies did not agree on which attentional processes involved even when using same tasks (Appendix C, available as supplemental digital content at http://links.lww.com/PAIN/B793). This could be partially explained by the specific methodological approach used or due to the different theoretical framework of interpretation. Furthermore, a careful interpretation is recommended concerning processes mentioned in different studies because of the various potential meanings used by different authors. As an alternative, an agreed global format would facilitate future research.

Third, we found that using words as a stimulus, either sensory or affective pain-related information, produced a larger effect than pictorial stimuli. This suggests that using words as a stimulus produces more potent effects than a pictorial stimulus, which is also aligned with the findings of the previous meta-analyses.^21,61^ However, it is worth noting that this finding might be driven by the greater use of words in the Stroop task, which also produced stronger effects, and that fewer studies used images overall. In addition, unlike most studies that focused on the sensory pain-related information and resulted in a small significant effect size, we found that the effect size for using affective pain-related information was moderate (Appendix E, available as supplemental digital content at http://links.lww.com/PAIN/B793). Yet, finding a consistent confirmation of attentional bias toward sensory pain-related information from 15 studies with relatively large sample sizes could help future studies using attentional bias-related approaches, such as recent studies trying to manage CP through using attentional bias modification (ABM) approach.^16^ Furthermore, as expected, including studies with larger sample size reflected in a relatively better-quality assessment outcomes comparing with previous meta-analyses (Appendix B, available as supplemental digital content at http://links.lww.com/PAIN/B793).

Fourth, the systematic review illustrates how different CP subtypes were linked with patterns of attentional processes. For instance, all studies that included fibromyalgia samples were found to have a faster reaction time toward pain-related information compared with the control group, which may reflect hypervigilance or facilitated attention (Table 3; Appendix C-Table 3A, 3B, and 3C, available as supplemental digital content at http://links.lww.com/PAIN/B793). Because this is an association and due to heterogeneity between samples recruited, it is not possible to know whether the differences between the patient groups are due to the type of task or stimuli being used or if these are genuine differences between CP subtype populations. Owing to the heterogeneity of the CP samples or low sample size used, we could only include the chronic lower back pain (ie, the most common CP) samples in the meta-analysis. More studies are needed to compare CP populations directly on the same type of task. This comparison would allow exploration of the possible links between the subtype of the CP populations and the correlated attentional processes. One possible explanation is that individuals with fibromyalgia have more widespread pain without an apparent physical cause, which triggers hypervigilance. This aligns with nociplastic pain mechanisms that explain the nature of fibromyalgia symptoms in light of the biopsychosocial perspective.^34^ Conversely, more localised CP that arises from a specific trigger (eg, arthritis and injury) is related to avoidance. Another possible reason could be that a broad range of pain words is more relevant for individuals with fibromyalgia than for people suffering from a specific pain problem. Although the main comorbid disorders were depression and anxiety, which linked to the development and maintenance of pain,^24^ the symptoms were subclinical in some studies suggesting an association rather than being causal factors in the development and maintenance of the CP. For instance, one study found that anxiety symptoms in individuals with fibromyalgia do not mediate the hypervigilance process.^37^ Furthermore, not all studies explored the comorbidity of anxiety and depression symptoms, so we were not able to examine comorbid symptoms in this review. For instance, the effect of depression on attention and other cognitive processes was explored in a previous systematic review and meta-analysis.^74^ Thus, we recommend future studies should examine and report how comorbid symptoms interact with CP and attentional processes. Furthermore, some studies showed that attentional biases among individuals with CP were related to daily activities and, thus, are likely to be linked to processes that maintain or exacerbate CP symptoms and disability.^83,89^ However, a previous meta-analysis did not find a link between CP and preattentive processes, which may not always be the case.^21^ Based on the current state of the art, there is no clear evidence that attentional biases are related to the level of disability in individuals with CP, the development of CP, or pain disability. Therefore, future studies are encouraged to test these relationships as well as potential solutions.

This is the first review that explored particularly the SA processes and their links to tasks, stimuli types, and interpretative models. Key strengths are the use of state-of-the-art systematic review and meta-analysis methods and the inclusion of studies with the most robust evidence by applying quality assessment criteria. However, there are also some limitations. Many studies were excluded from the meta-analysis because either they did not have a healthy control group (ie, We did not look at within-group bias), had a small sample size, including some recent eye-tracking studies that did not use goal-oriented reaction time, or the data reporting was poor. Poor data reporting was recognised across a considerable number of studies which did not improve after contacting study authors and, therefore, precluded the inclusion of these studies in the meta-analysis. For instance, one important additional meta-analysis would be examining the differences between congruent and incongruent reaction times. However, we could not do this comparison in the meta-analysis for 2 reasons; first, the number of papers that reported congruent–incongruent data was relatively small, and second, the studies that provided data used different equations to calculate the indices. Thus, using meta-analysis would result in a biased result. This could have been avoided if raw data were shared alongside the article, which aligns with the open science recommendations related to data sharing.^64^ We encourage future studies to use more standardised indices calculations that can be used in meta-analysis. In turn, having limitations in the ability to include studies in a meta-analysis decreases the ability to generalise the findings or produce more robust evidence about the attentional bias tasks, stimuli, or processes. Thus, we strongly recommend the use of open data. Moreover, the studies varied in their results considerably even when the same task and parameters (eg, dot-probe and cue duration equal 500 ms) were used, which did not allow us to explore the impact of additional factors such as time-points (Appendix C, available as supplemental digital content at http://links.lww.com/PAIN/B793). This would have been valuable to understand the dynamics of attention towards pain-related information. We recommend conducting an individual participant data meta-analysis to assess comprehensively various factors to detect differences in attentional biases between individuals with CP and healthy controls. Furthermore, when we included the studies in the meta-analysis and had a design with more than 2 groups, we chose the experimental group with the least comorbid symptoms to increase the homogeneity of the overall sample as much as possible. Finally, we tried to build the descriptive tables through careful extraction; however, the models and processes were not always presented clearly, due to reporting variations across papers.

## 5. Conclusion

This systematic review in the field of SA-CP explored reaction time-based studies with relatively large sample size and compared their different components descriptively. The meta-analysis confirmed that individuals with CP show a relatively small-to-moderate bias towards sensory pain-related information when conducting a meta-analysis through studies with large sample sizes. Furthermore, unlike previous reviews, we found that exploring studies with large sample size gave preliminary evidence that individuals with CP may show a moderate bias towards affective pain-related information. The evidence regarding attentional bias in people with CP was more substantial when using the Stroop task as well as word stimuli. However, more rigorous studies are still needed to gain robust evidence regarding attentional bias towards affective pain-related information. Exploring the main models that characterise attention among CP individuals can give a deeper understanding of the potential mechanisms around processes involved in the phenomena of their attentional biases. In addition, there are significant variations across the studies, which do not allow definitive conclusions about the role of types of stimuli and tasks, as well as whether these findings are valid across subgroups of individuals with CP. Overcoming such variation would help in comparing attentional processes found across different experiments. To do this, we strongly encourage open access data availability to overcome data reporting problems, enhance methodological quality, and enable universal use of meta-analysis in the future.

## Conflict of interest statement

The authors have no conflict of interest to declare.

## Appendix A. Supplemental digital content

Supplemental digital content associated with this article can be found online at http://links.lww.com/PAIN/B793.

## References

[1] Amaro-Díaz L Montoro CI Fischer-Jbali LR Galvez-Sánchez CM. Chronic pain and emotional stroop: a systematic review. J Clin Med 2022;11:3259. 35743329 10.3390/jcm11123259 PMC9224954

[2] Aronoff GM. Chronic pain and the disability epidemic. Clin J Pain 1991;7:330–8. 1809447 10.1097/00002508-199112000-00013

[3] Asmundson GJ Wright KD Hadjistavropoulos HD. Hypervigilance and attentional fixedness in chronic musculoskeletal pain: consistency of findings across modified stroop and dot-probe tasks. J Pain 2005;6:497–506. 16084464 10.1016/j.jpain.2005.02.012

[4] Baum C Schneider R Keogh E Lautenbacher S. Different stages in attentional processing of facial expressions of pain: a dot-probe task modification. J Pain 2013;14:223–32. 23294996 10.1016/j.jpain.2012.11.001

[5] Beck AT Clark DA. An information processing model of anxiety: automatic and strategic processes. Behav Res Ther 1997;35:49–58. 9009043 10.1016/s0005-7967(96)00069-1

[6] Beck JG Freeman JB Shipherd JC Hamblen JL Lackner JM. Specificity of Stroop interference in patients with pain and PTSD. J Abnormal Psychol 2001;110:536. 10.1037//0021-843x.110.4.536 11727943

[7] Berger A Henik A Rafal R. Competition between endogenous and exogenous orienting of visual attention. J Exp Psychol Gen 2005;134:207. 15869346 10.1037/0096-3445.134.2.207

[8] Bove VM Jr. Beyond images. Convergence 1996;2:30–46.

[9] Blicher A Reinholdt-Dunne ML Hvenegaard M Winding C Petersen A Vangkilde S. Engagement and disengagement components of attentional bias to emotional stimuli in anxiety and depression. J Exp Psychopathol 2020;11:2043808720943753.

[10] Broadbent P Liossi C Schoth DE. Attentional bias to somatosensory stimuli in chronic pain patients: a systematic review and meta-analysis. PAIN 2021;162:332–52. 32833792 10.1097/j.pain.0000000000002040

[11] Brown RJ Danquah AN Miles E Holmes E Poliakoff E. Attention to the body in nonclinical somatoform dissociation depends on emotional state. J Psychosomatic Res 2010;69:249–57. 10.1016/j.jpsychores.2010.04.010 20708447

[12] Brueggemann P Neff PK Meyer M Riemer N Rose M Mazurek B. On the relationship between tinnitus distress, cognitive performance and aging. Prog Brain Res 2021;262:263–85. 33931184 10.1016/bs.pbr.2021.01.028

[13] Brydges CR. Effect size guidelines, sample size calculations, and statistical power in gerontology. Innov Aging 2019;3:igz036. 31528719 10.1093/geroni/igz036 PMC6736231

[14] Bulcke CV Van Damme S Durnez W Crombez G. The anticipation of pain at a specific location of the body prioritizes tactile stimuli at that location. PAIN 2013;154:1464–8. 23707288 10.1016/j.pain.2013.05.009

[15] Carleton RN Richter AA Asmundson GJ. Attention modification in persons with fibromyalgia: a double blind, randomized clinical trial. Cogn Behav Ther 2011;40:279–90. 22060250 10.1080/16506073.2011.616218

[16] Carleton RN Asmundson GJ Korol SL LeBouthillier DM Hozempa K Katz JD Vlaeyen JWS Crombez G. Evaluating the efficacy of an attention modification program for patients with fibromyalgia: a randomized controlled trial. PAIN 2020;161:584–94. 31693540 10.1097/j.pain.0000000000001746

[17] Chan FH Suen H Jackson T Vlaeyen JW Barry TJ. Pain-related attentional processes: a systematic review of eye-tracking research. Clin Psychol Rev 2020;80:101884. 32585493 10.1016/j.cpr.2020.101884

[18] Cisler JM Bacon AK Williams NL. Phenomenological characteristics of attentional biases towards threat: a critical review. Cogn Ther Res 2009;33:221–234. 10.1007/s10608-007-9161-y PMC2901130 20622985

[19] Cohen J. Statistical power analysis for the behavioral sciences. Mahwah, NJ: Lawrence Earlbaum Associates, 1988; 20th Edition.

[20] Crombez G Van Damme S Eccleston C. Hypervigilance to pain: an experimental and clinical analysis. PAIN 2005;116:4–7. 15927387 10.1016/j.pain.2005.03.035

[21] Crombez G Van Ryckeghem DM Eccleston C Van Damme S. Attentional bias to pain-related information: a meta-analysis. PAIN 2013;154:497–510. 23333054 10.1016/j.pain.2012.11.013

[22] Darnall BD. Psychological treatment for patients with chronic pain. Washington, D.C.: American Psychological Association, 2019.

[23] Davis MC Zautra AJ Smith BW. Chronic pain, stress, and the dynamics of affective differentiation. J Pers 2004;72:1133–60. 15509279 10.1111/j.1467-6494.2004.00293.x PMC2570251

[24] de Heer EW Gerrits MM Beekman AT Dekker J Van Marwijk HW De Waal MW Spinhoven P Penninx BWJH Van Der Feltz-Cornelis CM. The association of depression and anxiety with pain: a study from NESDA. PLoS One 2014;9:e106907. 25330004 10.1371/journal.pone.0106907 PMC4198088

[25] Dear BF Sharpe L Nicholas MK Refshauge K. Pain-related attentional biases: the importance of the personal relevance and ecological validity of stimuli. J Pain 2011;12:625–32. 21310670 10.1016/j.jpain.2010.11.010

[26] Dear BF Sharpe L Nicholas MK Refshauge K. The psychometric properties of the dot-probe paradigm when used in pain-related attentional bias research. J Pain 2011;12:1247–54. 21982721 10.1016/j.jpain.2011.07.003

[27] den Heyer K Briand K Smith L. Automatic and strategic effects in semantic priming: an examination of Becker's verification model. Mem Cogn 1985;13:228–32. 10.3758/bf03197685 4046823

[28] Eccleston C Crombez G Aldrich S Stannard C. Attention and somatic awareness in chronic pain. PAIN 1997;72:209–15. 9272805 10.1016/s0304-3959(97)00030-4

[29] Eccleston C Crombez G. Pain demands attention: a cognitive–affective model of the interruptive function of pain. Psychol Bull 1999;125:356. 10349356 10.1037/0033-2909.125.3.356

[30] Eccleston C Crombez G. Worry and chronic pain: a misdirected problem solving model. PAIN 2007;132:233–6. 17961924 10.1016/j.pain.2007.09.014

[31] Egger M Smith GD Schneider M Minder C. Bias in meta-analysis detected by a simple, graphical test. BMJ 1997;315:629–34. 9310563 10.1136/bmj.315.7109.629 PMC2127453

[32] Fashler SR Katz J. More than meets the eye: visual attention biases in individuals reporting chronic pain. J Pain Res 2014;7:557. 25285022 10.2147/JPR.S67431 PMC4181742

[33] Fernández-Calderón F Lozano OM Moraleda-Barreno E Lorca-Marín JA Díaz-Batanero C. Initial orientation vs maintenance of attention: relationship with the severity of dependence and therapeutic outcome in a sample of cocaine use disorder patients. Addict Behav 2021;116:106834. 33503505 10.1016/j.addbeh.2021.106834

[34] Fitzcharles MA Cohen SP Clauw DJ Littlejohn G Usui C Häuser W. Nociplastic pain: towards an understanding of prevalent pain conditions. Lancet 2021;397:2098–110. 34062144 10.1016/S0140-6736(21)00392-5

[35] Franklin ZC Holmes PS Smith NC Fowler NE. Personality type influences attentional bias in individuals with chronic back pain. PLoS One 2016;11:e0147035. 26789517 10.1371/journal.pone.0147035 PMC4720440

[36] Giel KE Paganini S Schank I Enck P Zipfel S Junne F. Processing of emotional faces in patients with chronic pain disorder: an eye-tracking study. Front Psychiatry 2018;9:63. 29556205 10.3389/fpsyt.2018.00063 PMC5845113

[37] González JL Mercado F Barjola P Carretero I López-López A Bullones MA Fernández-Sánchez M Alonso M. Generalized hypervigilance in fibromyalgia patients: an experimental analysis with the emotional Stroop paradigm. J Psychosom Res 2010;69:279–87. 20708450 10.1016/j.jpsychores.2010.05.002

[38] Grisart JM Plaghki LH. Impaired selective attention in chronic pain patients. Eur J Pain 1999;3:325–33. 10700360 10.1053/eujp.1999.0138

[39] Haggman SP Sharpe LA Nicholas MK Refshauge KM. Attentional biases toward sensory pain words in acute and chronic pain patients. J Pain 2010;11:1136–45. 20797918 10.1016/j.jpain.2010.02.017

[40] Harbord RM Harris RJ Sterne JA. Updated tests for small-study effects in meta-analyses. Stata J 2009;9:197–210.

[41] Hayward DA Ristic J. Measuring attention using the Posner cuing paradigm: the role of across and within trial target probabilities. Front Hum Neurosci 2013;7:205. 23730280 10.3389/fnhum.2013.00205 PMC3656349

[42] Hedge C Powell G Sumner P. The reliability paradox: why robust cognitive tasks do not produce reliable individual differences. Behav Res Methods 2018;50:1166–86. 28726177 10.3758/s13428-017-0935-1 PMC5990556

[43] Higgins JP Thompson SG Deeks JJ Altman DG. Measuring inconsistency in meta-analyses. BMJ 2003;327:557–60. 12958120 10.1136/bmj.327.7414.557 PMC192859

[44] Ho AT Huynh KP Jacho-Chávez DT Rojas-Baez D. Data science in Stata 16: frames, lasso, and python integration. J Stat Softw 2021;98:1–9.

[45] Hoofs V Grahek I Boehler CN Krebs RM. Guiding spatial attention by multimodal reward cues. Atten Percep Psychophys 2022;84:655–70. 10.3758/s13414-021-02422-x 34964093

[46] Jones EB Sharpe L Andrews S Colagiuri B Dudeney J Fox E Heathcote LC Lau JYF Todd J Van Damme S Van Ryckeghem DML Vervoort T. The time course of attentional biases in pain: a meta-analysis of eye-tracking studies. PAIN 2021;162:687–701. 32960534 10.1097/j.pain.0000000000002083

[47] Kimmel M. Culture regained: Situated and compound image schemas. From perception to meaning: Image schemas in cognitive linguistics. Berlin: De Gruyter Mouton, 2005. p. 285–311.

[48] Köckerling F Simon T Adolf D Köckerling D Mayer F Reinpold W Weyhe D Bittner R. Laparoscopic IPOM versus open sublay technique for elective incisional hernia repair: a registry-based, propensity score-matched comparison of 9907 patients. Surg Endosc 2019;33:3361–9. 30604264 10.1007/s00464-018-06629-2 PMC6722046

[49] Kökönyei G Urbán R Reinhardt M Józan A Demetrovics Z. The difficulties in emotion regulation scale: factor structure in chronic pain patients. J Clin Psychol 2014;70:589–600. 24002923 10.1002/jclp.22036

[50] Lavy E Van den Hout M. Selective attention evidenced by pictorial and linguistic Stroop tasks. Behav Ther 1993;24:645–57.

[51] Lundh LG Eysenck MW. Anxiety: the cognitive perspective. Scand J Behav Ther 1994;23:61.

[52] Loeser JD Treede R-D. The Kyoto protocol of IASP basic pain terminology. PAIN 2008;137:473–7. 18583048 10.1016/j.pain.2008.04.025

[53] MacLeod C Mathews A Tata P. Attentional bias in emotional disorders. J Abnormal Psychol 1986;95:15. 10.1037//0021-843x.95.1.15 3700842

[54] Mahmoodi-Aghdam M Dehghani M Ahmadi M Banaraki AK Khatibi A. Chronic pain and selective attention to pain arousing daily activity pictures: evidence from an eye tracking study. Basic Clin Neurosci 2017;8:467. 29942430 10.29252/nirp.bcn.8.6.467 PMC6010654

[55] Mangun GR Hillyard SA. Mechanisms and models of selective attention. In: Rugg MD Coles MGH, editors. Electrophysiology of mind: event-related brain potentials and cognition. Oxford, United Kingdom; Oxford University Press, 1995. p. 40–85.

[56] Mazari A Derraz N. Language and culture. Int J Human Cult Stud 2015;2:350–9.

[57] Mazidi M Dehghani M Sharpe L Dolatshahi B Ranjbar S Khatibi A. Time course of attentional bias to painful facial expressions and the moderating role of attentional control: an eye-tracking study. Br J Pain 2019;15:5–15. 33633849 10.1177/2049463719866877 PMC7882769

[58] Mills SE Nicolson KP Smith BH. Chronic pain: a review of its epidemiology and associated factors in population-based studies. Br J Anaesth 2019;123:e273–83. 31079836 10.1016/j.bja.2019.03.023 PMC6676152

[59] Mirowski P. The future (s) of open science. Soc Stud Sci 2018;48:171–203. 29726809 10.1177/0306312718772086

[60] Mogg K Bradley B Miles F Dixon R. Brief report time course of attentional bias for threat scenes: testing the vigilance‐avoidance hypothesis. Cogn Emot 2004;18:689–700.

[61] Mogoaşe C David D Koster EH. Clinical efficacy of attentional bias modification procedures: an updated meta‐analysis. J Clin Psychol 2014;70:1133–57. 24652823 10.1002/jclp.22081

[62] Mohammadi S Dehghani M Sharpe L Heidari M Sedaghat M Khatibi A. Do main caregivers selectively attend to pain-related stimuli in the same way that patients do? PAIN 2012;153:62–7. 22001657 10.1016/j.pain.2011.08.021

[63] Moher D Liberati A Tetzlaff J Altman DG Group P. Preferred reporting items for systematic reviews and meta-analyses: the PRISMA statement. PLoS Med 2009;6:e1000097. 19621072 10.1371/journal.pmed.1000097 PMC2707599

[64] Munafò M. Open science and research reproducibility. Ecancermedicalscience 2016;10:ed56. 27350794 10.3332/ecancer.2016.ed56 PMC4898932

[65] Nguyen KN Watanabe T Andersen GJ. Role of endogenous and exogenous attention in task-relevant visual perceptual learning. PLoS One 2020;15:e0237912. 32857813 10.1371/journal.pone.0237912 PMC7454975

[66] O'Hara R Sharpe L Todd J. Cognitive biases among those with frequent or chronic headaches or migraines: a systematic review and meta-analysis. PAIN 2022;163:1661–9. 35067659 10.1097/j.pain.0000000000002554

[67] Page MJ McKenzie JE Bossuyt PM Boutron I Hoffmann TC Mulrow CD Shamseer L Tetzlaff JM Akl EA Brennan SE Chou R Glanville J Grimshaw JM Hróbjartsson A Lalu MM Li T Loder EW Mayo-Wilson E McDonald S McGuinness LA Stewart LA Thomas J Tricco AC Welch VA Whiting P Moher D. The PRISMA 2020 statement: an updated guideline for reporting systematic reviews. BMJ 2021;372:n71. 33782057 10.1136/bmj.n71 PMC8005924

[68] Pearce J Morley S. An experimental investigation of the construct validity of the McGill Pain Questionnaire. PAIN 1989;39:115–21. 2812848 10.1016/0304-3959(89)90182-6

[69] Peters ML Vlaeyen JW van Drunen C. Do fibromyalgia patients display hypervigilance for innocuous somatosensory stimuli? Application of a body scanning reaction time paradigm. PAIN 2000;86:283–92. 10812258 10.1016/S0304-3959(00)00259-1

[70] Pincus T Fraser L Pearce S. Do chronic pain patients ‘Stroop ‘on pain stimuli? Br J Clin Psychol 1998;37:49–58. 9547959 10.1111/j.2044-8260.1998.tb01278.x

[71] Pincus T Morley S. Cognitive-processing bias in chronic pain: a review and integration. Psychol Bull 2001;127:599. 11548969 10.1037/0033-2909.127.5.599

[72] Price-Williams D. Psychological experiment and anthropology: the problem of categories. Ethos 1974;2:95–114.

[73] Richards HJ Benson V Donnelly N Hadwin JA. Exploring the function of selective attention and hypervigilance for threat in anxiety. Clin Psychol Rev 2014;34:1–13. 24286750 10.1016/j.cpr.2013.10.006

[74] Rock PL Roiser J Riedel WJ Blackwell AD. Cognitive impairment in depression: a systematic review and meta-analysis. Psychol Med 2014;44:2029–40. 24168753 10.1017/S0033291713002535

[75] Roelofs J Peters ML Fassaert T Vlaeyen JW. The role of fear of movement and injury in selective attentional processing in patients with chronic low back pain: a dot-probe evaluation. J Pain 2005;6:294–300. 15890631 10.1016/j.jpain.2004.12.011

[76] Rusu AC Gajsar H Schlüter M-C Bremer Y-I. Cognitive biases toward pain. Clin J Pain 2019;35:252–60. 30499835 10.1097/AJP.0000000000000674

[77] Schmukle SC. Unreliability of the dot probe task. Eur J Pers 2005;19:595–605.

[78] Schoth DE Nunes VD Liossi C. Attentional bias towards pain-related information in chronic pain; a meta-analysis of visual-probe investigations. Clin Psychol Rev 2012;32:13–25. 22100743 10.1016/j.cpr.2011.09.004

[79] Snider BS Asmundson GJ Wiese KC. Automatic and strategic processing of threat cues in patients with chronic pain: a modified stroop evaluation. Clin J Pain 2000;16:144–54. 10870727 10.1097/00002508-200006000-00008

[80] Sterne JA Gavaghan D Egger M. Publication and related bias in meta-analysis: power of statistical tests and prevalence in the literature. J Clin Epidemiol 2000;53:1119–29. 11106885 10.1016/s0895-4356(00)00242-0

[81] Sterne JA Harbord RM. Funnel plots in meta-analysis. Stata J 2004;4:127–41.

[82] Thigpen NN Gruss LF Garcia S Herring DR Keil A. What does the dot‐probe task measure? A reverse correlation analysis of electrocortical activity. Psychophysiology 2018;55:e13058. 29314050 10.1111/psyp.13058 PMC5940518

[83] Todd J Sharpe L Johnson A Perry KN Colagiuri B Dear BF. Towards a new model of attentional biases in the development, maintenance, and management of pain. PAIN 2015;156:1589–600. 26291997 10.1097/j.pain.0000000000000214

[84] Todd J van Ryckeghem DM Sharpe L Crombez G. Attentional bias to pain-related information: a meta-analysis of dot-probe studies. Health Psychol Rev 2018;12:419–36. 30205757 10.1080/17437199.2018.1521729

[85] Van Damme S Legrain V Vogt J Crombez G. Keeping pain in mind: a motivational account of attention to pain. Neurosci Biobehav Rev 2010;34:204–13. 19896002 10.1016/j.neubiorev.2009.01.005

[86] Van Ryckeghem DM Crombez G Goubert L De Houwer J Onraedt T Van Damme SJP. The predictive value of attentional bias towards pain-related information in chronic pain patients: a diary study. PAIN 2012;154:468–75. 23375161 10.1016/j.pain.2012.12.008

[87] Van Ryckeghem DM Noel M Sharpe L Pincus T Van Damme S. Cognitive biases in pain: an integrated functional–contextual framework. PAIN 2019;160:1489–93. 30913168 10.1097/j.pain.0000000000001508

[88] Vlaeyen JW Kole-Snijders AM Rotteveel AM Ruesink R Heuts PH. The role of fear of movement/(re) injury in pain disability. J Occup Rehabil 1995;5:235–52. 24234727 10.1007/BF02109988

[89] Vlaeyen JW Linton SJ. Fear-avoidance and its consequences in chronic musculoskeletal pain: a state of the art. PAIN 2000;85:317–32. 10781906 10.1016/S0304-3959(99)00242-0

[90] Vlaeyen JW Morley S Crombez G. The experimental analysis of the interruptive, interfering, and identity-distorting effects of chronic pain. Behav Res Ther 2016;86:23–34. 27614948 10.1016/j.brat.2016.08.016

[91] Yang Z Jackson T Chen H. Effects of chronic pain and pain-related fear on orienting and maintenance of attention: an eye movement study. J Pain 2013;14:1148–57. 23850178 10.1016/j.jpain.2013.04.017

